# Visual Acuity and Number of Amniotic Membrane Layers as Indicators of Efficacy in Amniotic Membrane Transplantation for Corneal Ulcers: A Multicenter Study

**DOI:** 10.3390/jcm10153234

**Published:** 2021-07-22

**Authors:** Javier Lacorzana, Antonio Campos, Marina Brocal-Sánchez, Juan Marín-Nieto, Oswaldo Durán-Carrasco, Esly C. Fernández-Núñez, Andrés López-Jiménez, Jose L. González-Gutiérrez, Constantinos Petsoglou, Jose L. García Serrano

**Affiliations:** 1 Department of Ophthalmology, Virgen de las Nieves University Hospital, 18006 Granada, Spain; 2 Doctoral Program in Clinical Medicine and Public Health, University of Granada, 18006 Granada, Spain; 3 Tissue Engineering Group, Department of Histology, University of Granada, 18006 Granada, Spain; acampos@ugr.es; 4 Institute of Biosanitary Research ibs. Granada, University of Granada, 18006 Granada, Spain; 5 Department of Ophthalmology, Son Espases University Hospital, 07120 Palma de Mallorca, Spain; mabroc2@hotmail.com; 6 Department of Ophthalmology, Virgen de la Victoria University Hospital, 29010 Malaga, Spain; juan7_m@hotmail.com; 7 Department of Ophthalmology, Nuestra Señora de la Candelaria University Hospital, 38010 Santa Cruz de Tenerife, Spain; oswaldurancarrasco@gmail.com (O.D.-C.); fernandez_729@hotmail.com (E.C.F.-N.); 8 Department of Ophthalmology, Reina Sofía University Hospital, 30003 Murcia, Spain; andreslj_2005@hotmail.com; 9 Department of Ophthalmology, Juan Ramón Jimenez, University Hospital, 21005 Huelva, Spain; joselgogu@hotmail.com; 10 Department of Ophthalmology, Sydney Eye Hospital, Sydney 2100, Australia; conpetsoglou@hotmail.com; 11 Department of Ophthalmology, San Cecilio University Hospital, 18006 Granada, Spain; jopalace@hotmail.com

**Keywords:** amniotic membrane, amniotic membrane transplantation, cornea, corneal ulcer, corneal ulceration, non healing corneal ulcer, visual acuity, persistent epithelial defects

## Abstract

Background: To evaluate new indicators in the efficacy of amniotic membrane transplantation (AMT) for non-healing corneal ulcers (NHCUs). Methods: Retrospective, multicenter study. In total, 223 AMTs for NHCU in 191 patients were assessed. The main outcomes studied were the success rate of AMT (complete re-epithelization), postoperative visual acuity (VA) gain, and number of AM layers transplanted. Results: The overall AMT success rate was 74.4%. In 92% of our patients VA stability or improvement. Postoperative VA was significantly higher than preoperative VA in the entire cohort (*p* < 0.001) and in all etiological groups of ulcers (post-bacterial, *p* ≤ 0.001; post-herpetic, *p* ≤ 0.0038; neurotrophic ulcers, *p* ≤ 0.014; non-rheumatic peripheral, *p* ≤ 0.001; and ulcers secondary to lagophthalmos and eyelid malposition or trauma, *p* ≤ 0.004). Most participants (56.5%) presented a preoperative VA equal to or less than counting fingers (≤0.01). Of these, 13.5% reached a postoperative VA equal to or better than legal blindness (≥0.05) after AMT. A higher success rate was observed in the monolayer than in the multilayer AMT (79.5% and 64.9%, respectively; *p* = 0.018). No statistically significant values were found between the number of layers transplanted and VA gain (*p* = 0.509). Conclusion: AMT is not only beneficial in achieving complete re-epithelialization in NHCUs but also in improving postoperative VA; these improvements are independent of etiologies of ulcers. Furthermore, the use of monolayer AMT seems to be a more appropriate option than multilayer AMT for NHCU since the multilayer AMT did not present better outcomes (success rate and VA gain) compared to monolayer AMT in the different types of ulcers studied.

## 1. Introduction

The cornea is a body surface exposed to the external environment. The protective factors of the cornea include the eyelids and the tear film among others. The latter is responsible for nourishing the avascular cornea while providing a stable refractive surface.

A stable tear film and an integrated corneal surface are of great importance for good visual acuity (VA) [1,2]. Different authors [2,3,4] have studied the histological and immunohistochemical methods for the alterations of the tear film and the cornea and their implication in VA.

A non-healing corneal ulcer (NHCU) is defined as an ulcer that does not show any indication of complete corneal epithelialization within two weeks despite the administration of proper medical treatment [5]. It can be caused by multiple conditions such as neurotrophic keratitis, infection, rheumatic disease, eyelid malposition, trauma, and corneal dystrophy [5,6]. NHCUs can progress to descemetoceles or perforations, which is why their rapid treatment is highly recommended. In this regard, amniotic membrane transplantation (AMT) has been suggested as an excellent therapeutic option [5,6].

The amniotic membrane (AM) is the innermost layer of the placenta. It is a thin (20-500 µm), semi-transparent membrane. Histologically, the AM comprises three layers: epithelium, basement membrane, and avascular stroma [7,8,9,10,11,12,13].

This tissue has multiple biological properties, including the induction of cell proliferation, reduction of neovascularization, anti-scarring properties, increasing migration of cells such as keratinocytes, pool corneal regeneration, and little or no immunogenicity [7,14,15,16,17,18,19,20]. Heavy chain hyaluronan/pentraxin 3 (HC-HA/PTX3), a matrix component of AM, is a key factor responsible for the aforementioned AM’s properties [16,17,21]. These properties favor its use in ophthalmological pathologies, such as persistent corneal ulcers (neurotrophic, post-herpetic) [7,22,23,24,25,26,27,28], descemetoceles [29], perforations [7,14,26,30,31,32], and chemical burns [7,14,33,34,35,36,37,38,39].

Although the usefulness of AMT in treating NHCU has previously been investigated, several questions remain unanswered, especially with regard to VA gain and the number of layers used [6,31,40,41,42]. The current uncertainties may be due to the differences in the etiology of ulcers, insufficient statistical approach, or indicators evaluated [5,6,31,40,42,43]. In order to address these limitations, the present multicenter study sought to assess the efficacy of AMT in a large sample of patients with NHCUs while considering the VA gain and number of layers used. In addition, we aimed to explore these effects on different etiological groups and the possible influence of numbers of layers used in VA gain, a novel approach to be considered [6,40].

## 2. Materials and Methods

### 2.1. Patients

This retrospective multicenter study was conducted to evaluate the efficacy of AMT in treating NHCUs at Spanish National Healthcare hospitals. Patient records from January 2012 to June 2018 were obtained. Our study covered 17 participating hospitals throughout Spain. The patient population from these hospitals represents 13.78% (6,436,043/46,720,000) of the Spanish population. Written informed consent was obtained from all patients prior to the transplantation. This study was approved by the local Ethics Committee (Study code: 1272-N-18) and adhered to the tenets of the Declaration of Helsinki.

**Figure 1:**
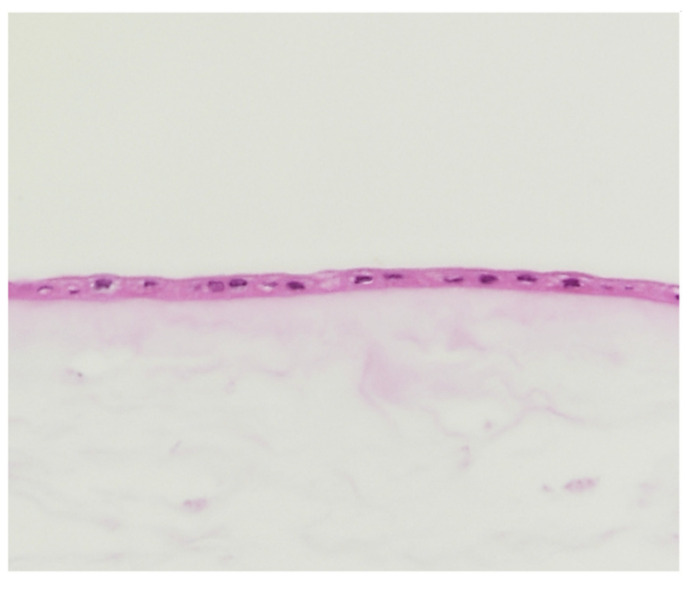
Amniotic membrane (×20) stained with *hematoxylin and eosin* provided by Biobank. Epithelium and avascular stroma.

We analyzed data from 223 AMT cases in 191 patients with NHCUs of different etiologies. In all cases, cryopreserved AMs were used as they contain high concentrations of growth factors [7,44] and lubricin, a boundary lubricant (Figure 1) [17,45]. Cryopreserved AMs are most widely used because of their biosafety [43] The protocols of provincial and regional biobanks established by the National Health System were followed, with sterility controls and serological studies of donors and recipients [15].

Patients with corneal ulcers refractory to medical treatment, surgically treated with cryopreserved AM, were included. Only patients with a minimum follow-up of 18 months post-surgery were included in accordance with the inclusion criteria. The ulcers were categorized as post-bacterial ulcers, post-herpetic ulcers, neurotrophic ulcers, peripheral corneal ulcers not associated with a rheumatic disease, and ulcers caused by lagophthalmos, eyelid malposition, or trauma [6,40,43]. The exclusion criteria were as follows: inability to follow-up, incomplete records, and coadjutant surgery (e.g., conjunctival flap, tarsorrhaphy, or lamellar keratoplasty). Incomplete or unclear records were evaluated by two investigators with expertise (LJ and GSJL). In cases of disparity, the patient was excluded. NHCUs with an active infection, descemetocele, or perforation were excluded. Furthermore, patients with ulcers due to bullous keratopathy, post-keratoplasty ulcers, rheumatic corneal ulcers, stem cell deficiencies (requiring different surgical techniques), or chemical burns (requiring AMT within two weeks, thus not qualified as an NHCU) were excluded [5,46]. The measurement of ulcers’ width was not standardized at all centers, which is why this parameter was excluded from our study. An identity document (ID) was assigned to each center, and each AMT used for treatments received an individualized ID. The outcome variable was the success or failure of the surgery.

Success was defined as the complete epithelialization of the refractory corneal ulcer eight weeks after surgery (lack of fluorescein staining at the slit lamp examination). Confocal microscopy studies have shown that AM may be present up to six weeks, and it might not be detectable eight weeks after surgery [47] AMT failure was defined as incomplete corneal epithelization within eight weeks after intervention. If two or more AMTs were performed, the results were analyzed. If other types of reconstructive surgery were performed post-AMT, the results were censored at this time [40].

The independent variables collected in each case included the following: sex, age, number of AMTs performed in each patient, number of AM layers used in each AMT, etiology of the ulcers, whether AMT was the primary surgical option or not, VA before transplantation, VA after the last follow-up, and corneal transparency after AMT. The corneal opacification was based on the Sotozono classification [48]: transparent (grade 0), partially opaque (grades 1 and 2), and opaque (grade 3) corneas.

VA was evaluated in all patients using the Snellen’s original test with conversions to decimal and logMAR scales [49,50] for statistical analyses. Lower VAs were calculated as follows: counting fingers, 1/100 (logMAR 2); hand motions, 1/200 (logMAR 2.3); light perception, 1/666 (logMAR 2.8) [51], and amaurosis, 0 (logMAR 3).

### 2.2. Surgery and Follow-Up

All surgeries were performed by consultant ophthalmologists (n = 21). To homogenize their results, the following quality criteria were required: fill in a single and unified questionnaire, clearly defined ulcer type, definition of success or failure of AMT, performed in public hospitals with training program in ophthalmology, and surgery performed by ocular surface specialists with more than five years of experience.

The cryopreserved AMs were prepared according to the Tseng method [15]. The surgeons obtained the AMs from the regional tissue banks maintained by the Spanish government. All patients underwent a thorough preoperative examination. Surgery was performed under topical, peribulbar, or general anesthesia, as determined by the surgeon. Prior to the AMT, necrotic edges of the ulcer were debrided. Depending on the severity, the AMs were applied as a monolayer or as multilayers (≥2), fixed with interrupted or uninterrupted 10-0 nylon sutures or fibrin sealants. The AMs could be transplanted using different techniques: (1) inlay—it was placed over the ocular defect without extending beyond its edges; (2) overlay—it was used as a patch suturing it beyond the edges of the ocular defect; and (3) a combination of both methods, known as the “sandwich” technique [7,40].

Postoperatively, the patients received treatment with antibiotics and topical corticosteroids, in addition to the etiological treatment for the ulcer. Follow-up examinations were performed the day after, and approximately one, two, four, and eight weeks after the operation. The patients were treated in the emergency department in case of any complications. Subsequent follow-ups were performed at the discretion of the physician for at least 18 months. When AMT was not the primary surgical option, the alternative options were conjunctival flap, tarsorrhaphy, and lamellar or penetrating keratoplasty [23,52].

### 2.3. Statistical Analysis

Statistical analysis was performed using the SPSS Statistics 19 software (IBM Corp., Armonk, NY, USA). The numerical variables are expressed as means ± standard deviations. The categorical variables are described as absolute (n) and relative (%) frequencies. For nonparametric data distribution, a Mann-Whitney test was used. Odds ratios (ORs) were calculated for variables related to success, along with their 95% confidence intervals (CIs). For differences between the types of ulcers, a Kruskal-Wallis test was applied. VA data were normalized to the logMAR scale, and a Wilcoxon signed-rank test was applied to analyze the relationship between the VAs before and after the intervention. These values were also expressed on the Snellen optotype scale. A *p*-value < 0.05 was considered statistically significant.

For the determination of the sample size, 34 AMTs would be needed in each of the 5 subgroups (n = 170); accepting an alpha risk of 0.005 and beta risk of 0.2 in a two-sided test, and estimating the significant post-AMT VA gain at 0.3 logMAR and the variance at 0.8. Furthermore, an exhaustive review of the literature on this subject was carried out. It was found that the studies with the largest sample size and best design were those of Schuerch et al. [6] and Uhlig et al. [40] (149 and 108 patients, respectively). In our article, we analyzed data from 191 patients (223 AMT cases).

## 3. Results

**Figure 2:**
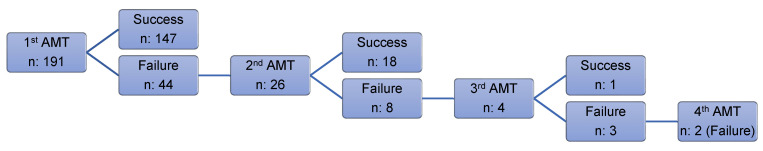
Flowchart of success and failure after amniotic membrane transplantation.

In our study, we analyzed 223 AMTs that were used in 191 patients (94 male and 97 female patients) with NHCUs of different etiologies (Figure 2).

### 3.1. Sex and Age

Sex distribution analysis revealed that 46.6% (n = 104) of the 223 AMTs were performed in male patients and 53.4% (n = 119) in female patients (Table 1). The mean age of patients receiving an AMT was 65 ± 18.3 years (range 11–102 years) (Table 1).

### 3.2. Success and Failure

**Figure 3:**
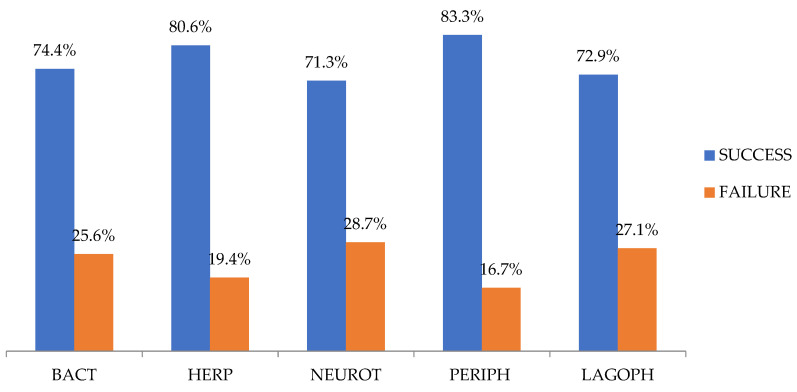
Amniotic membrane transplantation success and failure rates for each group of corneal ulcers. BACT, post-bacterial ulcers; HERP, post-herpetic ulcers; NEUROT, neurotrophic ulcers; PERIPH, non-rheumatic peripheral ulcers; LAGOPH, ulcers secondary to lagophthalmos and eyelid malposition or trauma.

Of all the AMTs, 74.4% (166/223) were successful, and there were no statistically significant differences among the NHCU cases of various etiologies (*p* = 0.755) (Figure 3).

### 3.3. Corneal Opacification

Since these were refractory ulcers secondary to a serious corneal pathology, transparency was reduced at the beginning. The cornea was transparent after AMT (grade 0 classification Sotozone) in 22.4% (51/223), partially opaque (grades 1 and 2) in 18.8% (42/223), and opaque (grade 3) in 58.3% (130/223) (Table 1).

### 3.4. Visual Acuity

**Figure 4:**
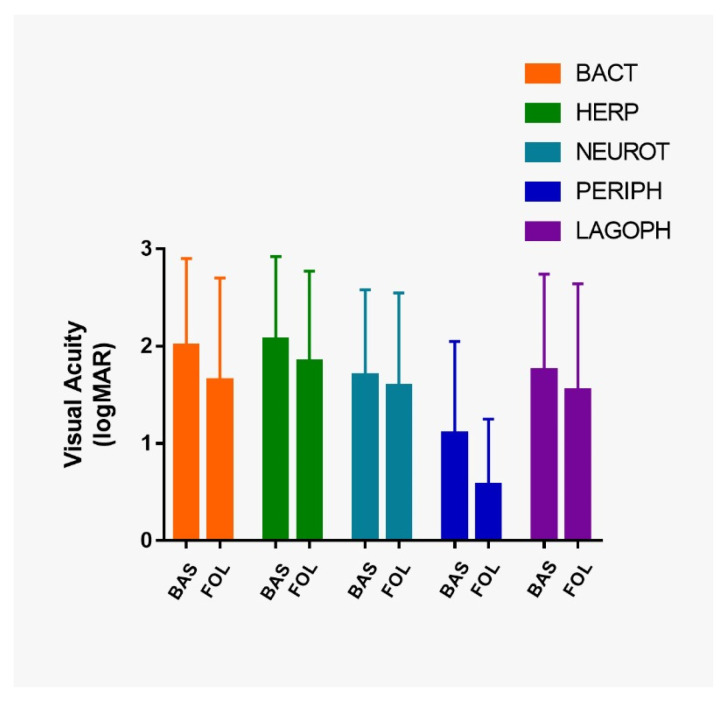
Bar chart representing visual acuity (VA) in logMAR at baseline and at the last follow-up. VA, visual acuity; BACT, post-bacterial ulcers; HERP, post-herpetic ulcers; NEUROT, neurotrophic ulcers; PERIPH, non-rheumatic peripheral ulcers; LAGOPH, ulcers secondary to lagophthalmos and eyelid malposition or trauma; BAS, at baseline; FOL, at the last follow-up.

Preoperative VA was significantly different in each type of ulcer (*p* = 0.001). Preoperative VA was significantly worse in post-herpetic ulcers compared to those in neurotrophic ulcers (*p* = 0.03), and non-rheumatic peripheral ulcers (*p* = 0.003) (Figure 4).

In the entire study population, the VA significantly improved from 1.77 ± 0.93 to 1.54 ± 1.02 logMAR (*p* < 0.001). Postoperative VA was significantly higher than preoperative VA in all etiological groups of ulcers: post-bacterial ulcers (*p* ≤ 0.001), post-herpetic ulcers (*p* ≤ 0.0038), neurotrophic ulcers (*p* ≤ 0.014), non-rheumatic peripheral corneal ulcers (*p* ≤ 0.001) and in ulcers secondary to lagophthalmos and eyelid malposition or trauma (*p* ≤ 0.004) (Figure 4).

VA stability or improvement was seen in 92% (205/223) of the cases (Table 2). The participants presented a preoperative VA equal to or less than counting fingers (≤0.01) in 56.5% of the study population (126/223). Of those, 13.5% (17/126) reached a postoperative VA equal to or better than legal blindness (≥0.05) due to AMT [53,54].

### 3.5. Efficacy of Monolayer Versus Multilayer AMT

Monolayer and multilayer AMTs were performed in 65.5% (146/223) and 34.5% (77/223) of the cases, respectively. The success rate was higher in the monolayer (79.5%) than in the multilayer AMTs (64.9%) (*p* = 0.018) (Table 3).

### 3.6. Correlation of Monolayer/Multilayer AMT and VA Gain

In the monolayer and multilayer AMT, the VA improved from 2.04 ± 0.88 to 1.80 ± 1.01 logMAR (*p* = 0.093) and 1.49 ± 0.91 to 1.28 ± 0.97 logMAR (*p* = 0.056), respectively. Table 4 shows the relationship between the number of amniotic membrane layers and VA gain according to ulcer etiology (Table 4).

There were no statistically significant differences in VA gain between the monolayer and multilayer AMT in the entire cohort (*p* = 0.509) or in all etiological groups (post-bacterial ulcers, *p* = 0.208; post-herpetic ulcers, *p* = 0.338; neurotrophic ulcers, *p* = 0.737; non-rheumatic peripheral ulcers, *p* = 0.054; ulcers secondary to lagophthalmos and eyelid malposition or trauma, *p* = 0.371).

## 4. Discussion

Several studies have described the benefits of AMTs as a treatment for various ocular surface pathologies [14,34,35,55], including NHCUs [56,57,58]. However, the diverse etiologies of the ulcers, the small sample sizes, and the adjuvant treatments used or the analysis of the different variables make direct comparisons difficult [5,6,31,40,42,43]. Our multicenter study aimed to study a series of indicators in a large sample size, with a special emphasis on VA gain and number of layers. Thus, we sought to assess and confirm ideas related to the effectiveness of the use of AMT, all the while suggesting new ones to reinforce its use.

The mean age of the participants in our study and other studies were between 64 and 68 years [6,40,51,59]. We found that AMT was a safe and non-aggressive technique and so can be used in a wide range of ages, a fact that was also supported by other studies [6,40,59]. Therefore, in the absence of a response to pharmacological treatment, AMT became the first surgical option for NHCU in the hospitals enrolled in our study. This idea was also supported by a recently published article that revealed that corneal ulcers are the first indications for AMTs [60].

Our overall percentage of success was 74.4% (166/223). Success rates of AMT on the ocular surface are highly variable, ranging from 49% to 97–100% [6,40,43,57]. Success rates as high as 49% (66/135) and 70% (105/149) have been reported by Uhlig et al. [40] and Schuerch et al. [6], respectively. The success rate in our study is consistent with that observed in these studies, with a comparatively larger sample size. Nevertheless, when comparing the results of these studies, the fact that the NHCU cases treated with AMT did not have a homogeneous etiological classification should be considered. Schuerch et al. [6] studied the same etiologies as the ones studied here. Moreover, they focused on other etiologies, such as post-keratoplasty associated with rheumatic disease, secondary to bullous keratopathy, and ulcers due to chemical burns. Uhlig et al. [40] divided NHCU cases into four groups (neurotrophic, post-herpetic, post-bacterial, and rheumatologic). Among their examined groups, there were no significant differences in the epithelization percentages. Liu et al. [43] also reported no significant differences between their infectious and non-infectious ulcer groups. In line with the results reported above, no significant differences were observed in the percentages of AMT success (71.3% to 83.3%) among our five groups of corneal ulcers.

Preoperative VA was very low throughout the cohort. Schuerch et al. [6] observed no difference in the preoperative VA between different etiological groups. However, we found that preoperative VA was significantly worse in the group with post-herpetic ulcers compared to the other NHCU etiological groups. Post-herpetic ulcers presented a high percentage of opacification, even though they were well re-epithelialized. Although AMT is an effective technique for the closure of these ulcers in many cases, the initial treatment of the post-herpetic ulcers should be faster since the loss of transparency was more frequent than in other ulcers. Therefore, despite the healing that was achieved, the VA at the last follow-up was low in most cases.

In contrast to our postoperative VA findings, Uhlig et al. [40], Letko et al. [41], Prabhasawat et al. [31], and Brocks et al. [61] did not find significant improvements in postoperative VA compared to the baseline values despite reporting high percentages of re-epithelization. Schuerch et al. [6] observed significant improvements in VA in the entire cohort but not in the etiological groups. In our study, we found significant improvements in VA across the entire cohort and also in all the etiological groups. Thus, since all groups improved, the etiology of ulcers does not seem to be a decisive factor in the VA improvements. Although, these improvements were small in some cases, even these small VA gains are relevant in the daily lives of patients with such low VA. Moreover, this gain may allow some of them to stop being blind (13.5%, 17/126), with all that this visual improvement entails clinically, as we have been able to observe in our results. HC-HA/PTX3’s anti-scarring and anti-angiogenic effects of AM could help explain the VA improvements [16,21,62].

A recent study using self-retained cryopreserved AMs (Prokera^®^; Bio-Tissue, Inc., Miami, FL, USA) has shown significant VA gain in a limited number of cases (n = 24); however, this study only analyzed Prokera^®^ in infectious ulcers but not in NHCUs. Others studies on Prokera^®^ did not find this significant VA gain [61].

A total of 92% (205/223) of our patients maintained or improved their VA scores. This result made us consider several ideas. First, vascular progression and persistent inflammation often facilitate the closure of ulcers, with secondary consequence being the loss of corneal transparency and reduction in VA. AMTs could help avoid this by self-integration into the host corneal tissue in different patterns (subepithelial, intraepithelial, or intrastromal) [63,64]. However, if we use it later, there may already be a certain component of fibrotic stroma that reduces corneal transparency in a pronounced way. The initial treatment of these chronic ulcers must be aggressive and rapid to prevent the loss of initial VA; otherwise, only a low percentage of patients will have moderate vision restored. Hence, we strongly recommend that AMT should be used as soon as possible. Second, AMTs can improve VA by regularizing the corneal surface and improving the transparency of the cornea [41,65]; this may delay the employment of more aggressive surgical options. In the future, many of these patients could receive a corneal transplant since their cornea will have been epithelialized and will be less inflamed [23,26,43].

With regard to AM layers and the success rate, there are two previous studies that included a relatively low number of cases (n = 28 in each study) and reported that the success rate of monolayer AMT ranged from 64% to 80% and that of multilayer AMT ranged from 72% to 84.6% [31,42]. In our multicenter study, the success rate was significantly higher with the monolayer AMT than with the multilayer AMT (79.5% and 64.9%, respectively). This result must be cautiously interpreted as the ulcers’ depth could be analyzed (descemetoceles were discarded) but not the ulcers’ width. Perhaps, larger ulcers lead surgeons to apply more layers in order to try achieving greater success or this may depend on the surgeons’ preferences. Another possible hypothesis is that AM does not integrate well when used as a multilayer. This hypothesis could be evaluated at the level of optical microscopy in future studies.

Regarding the influence of the number of AM layers on VA gain, our results showed that there were no significant differences between monolayer and multilayer AMT in all etiological groups.

Thus, since the success rate and VA gain were not better in multilayer AMT than in monolayer AMT, the use of multilayer AMT could not be justified in NHCU. Nonetheless, the use of multilayer AMT could be indicated in specific cases such as descemetoceles and perforations [19,26,27,63,64].

A limitation to our study could be its retrospective and multicenter nature. AM availability, surgeons’ therapeutic preferences, and prevalence of ulcers could have conditioned the selection of the different cases in our studied hospitals. Possibly, the assessment of the ulcers’ width would have helped to better explain our results; however, these data were not consistent and, thus, were excluded from our study.

In conclusion, this multicenter study has assessed VA gain and the number of AM layers transplanted as new indicators in the evaluation of NHCUs. Our results revealed not only that the use of AMT is beneficial in achieving complete re-epithelialization in NHCUs but also that it improves the postoperative VA independent of the etiology of the ulcers. Moreover, we demonstrated that the use of multilayer AMT in NHCUs does not improve the outcomes (success rate and VA gain) in comparison to monolayer AMT in the different types of ulcers studied. In addition, to the best of our knowledge, this is the largest sample size included in a study evaluating the efficacy of AMT as a treatment for patients with NHCUs [6,40].

## Figures and Tables

**Table 1:** Amniotic membrane transplantation for each type of ulcer.

	BACT	HERP	NEUROT	PERIPH	LAGOPH
	N = 39	N = 31	N = 87	N = 18	N = 48
	−17.50%	−13.90%	−39.00%	−8.10%	−21.50%
Sex					
Male	21 (53.8%)	16 (51.6%)	36 (41.4%)	9 (50%)	22 (45.8%)
Female	18 (46.2%)	15 (48.4%)	51 (58.6%)	9 (50%)	26 (54.2%)
Age	66.4 ± 16.9	63.6 ± 15.5	66.4 ± 17.8	65.4 ± 16.9	61.9 ± 22.4
(mean ± SD)					
1^a^ surgical option	32 (82.1%)	24 (77.4%)	73 (83.9%)	14 (77.8%)	42 (87.5%)
Corneal opacification					
Grade 0	9 (23.1%)	1 (3.2%)	17 (19.5%)	12 (66.7%)	12 (25%)
Grade 1–2	6 (15.4%)	1 (3.2%)	24 (27.6%)	2 (11.1%)	9 (18.8%)
Grade 3	24 (61.5%)	29 (93.5%)	46 (52.9%)	4 (22,2%)	27 (56.2%)

BACT, post-bacterial ulcers; HERP, post-herpetic ulcers; NEUROT, neurotrophic ulcers; PERIPH, non-rheumatic peripheral ulcers; LAGOPH, ulcers secondary to lagophthalmos and eyelid malposition or trauma; SD, standard deviation; 1^a^ surgical option, primary surgical option.

**Table 2:** Functional results (visual acuity) of amniotic membrane transplantation for each type of ulcer. The minimum last follow-up was 18 months.

	BACT	HERP	NEUROT	PERIPH	LAGOPH
VA at baseline					
- Amaurosis	6 (15.4%)	1 (3.2%)	3 (3.4%)	1 (5.6%)	2 (4.2%)
- LP	7 (17.9%)	12 (38.8%)	13 (14.9%)	2 (11.1%)	16 (33.3%)
- HM	10 (25.6%)	5 (16.1%)	22 (25.3%)	1 (5.6%)	6 (12.5%)
- CF	4 (10.3%)	5 (16.1%)	10 (11.5%)	0 (0%)	0 (0%)
- ≥0.05 a 1 Snellen	12 (30.8%)	8 (25.8%)	39 (44.8%)	14 (77.8%)	24 (50%)
VA at last follow-up					
- Amaurosis	6 (15.4%)	1 (3.2%)	4 (4.6%)	1 (5.5%)	2 (4.2%)
- LP	5 (12.8%)	8 (25.8%)	11 (12.6%)	0 (0%)	14 (29.2%)
- HM	7 (17.9%)	5 (16.1%)	19 (21.9%)	0 (0%)	5 (10.4%)
- CF	1 (2.6%)	7 (22.7%)	13 (14.9%)	0 (0%)	1 (2.1%)
- ≥0.05 a 1	20 (51.3%)	10 (32.3%)	40 (46%)	17 (94.5%)	26 (54.2%)
Changes in VA					
Worsening	1 (2.6%)	5 (16.1%)	8 (9.2%)	1 (5.6%)	3 (6.3%)
Equal	22 (56.4%)	15 (48.4%)	54 (62.1%)	2 (11.1%)	30 (62.5%)
Improvement	16 (41%)	11 (35.5%)	25 (28.7%)	15 (83.3%)	15 (31.3%)

VA, visual acuity; BACT, post-bacterial ulcers; HERP, post-herpetic ulcers; NEUROT, neurotrophic ulcers; PERIPH, non-rheumatic peripheral ulcers; LAGOPH, ulcers secondary to lagophthalmos and eyelid malposition or trauma; CF, counting fingers; HM, hand motions; LP; light perception.

**Table 3:** Relationship between the number of AM layers and success rate according to ulcer etiology.

	BACT	HERP	NEUROT	PERIPH	LAGOPH
MON	88.9%	90.9%	75.0%	77.8%	75.7%
MUL	61.9%	55.6%	63.0%	88.9%	63.6%
TOTAL	74.4%	80.6%	71.3%	85.3%	72.9%
p	*p* = 0.058	*p* = 0.043	*p* = 0.186	*p* = 0.500	*p* = 0.334

MON, monolayer; MUL, multilayer; BACT, post-bacterial ulcers; HERP, post-herpetic ulcers; NEUROT, neurotrophic ulcers; PERIPH, non-rheumatic peripheral ulcers; LAGOPH, ulcers secondary to lagophthalmos and eyelid malposition or trauma.

**Table 4:** Relationship between the number of amniotic membrane layers and visual acuity gain according to ulcer etiology.

		BACT	HERP	NEUROT	PERIPH	LAGOPH
**MON**	logMAR VA at baseline (mean ± SD)	2.19 ± 0.93	2.20 ± 0.79	1.89 ± 0.87	1.56 ± 1.05	2.21 ± 0.86
	logMAR VA last follow-up (mean ± SD)	1.88 ± 1.14	1.84 ± 0.97	1.82 ± 0.94	0.61 ± 0.33	2.11 ± 0.97
**MUL**	logMAR VA at baseline (mean ± SD)	1.69 ± 0.79	1.81 ± 0.94	1.48 ± 0.85	0.81 ± 0.80	1.52 ± 0.98
	logMAR VA last follow-up (mean ± SD)	1.28 ± 0.79	1.84 ± 0.89	1.35 ± 0.93	0.55 ± 0.85	1.25 ± 1.05

VA, visual acuity; MON, monolayer; MUL, multilayer; BACT, post-bacterial ulcers; HERP, post-herpetic ulcers; NEUROT, neurotrophic ulcers; PERIPH, non-rheumatic peripheral ulcers; LAGOPH, ulcers secondary to lagophthalmos and eyelid malposition or trauma; SD, standard deviation.

## Data Availability

The data presented in this study are available on request from the corresponding author. The data are not publicly available due to restrictions of privacy.
